# Mechanical loading due to muscle movement regulates establishment of the collagen network in the developing murine skeleton

**DOI:** 10.1098/rsos.231023

**Published:** 2023-10-18

**Authors:** Saima Ahmed, Andrew V. Rogers, Niamh C. Nowlan

**Affiliations:** ^1^ Department of Bioengineering, Imperial College London, London, UK; ^2^ Electron Microscopy Unit, Royal Brompton Hospital, London, UK; ^3^ School of Mechanical and Materials Engineering, University College Dublin, Dublin, Ireland; ^4^ UCD Conway Institute, University College Dublin, Dublin, Ireland

**Keywords:** collagen I, collagen II, collagen VI, collagen X, collagen XI, bioreactor

## Abstract

Mechanical loading is critical for collagen network maintenance and remodelling in adult skeletal tissues, but the role of loading in collagen network formation during development is poorly understood. We test the hypothesis that mechanical loading is necessary for the onset and maturation of spatial localization and structure of collagens in prenatal cartilage and bone, using *in vivo* and *in vitro* mouse models of altered loading. The majority of collagens studied was aberrant in structure or localization, or both, when skeletal muscle was absent *in vivo*. Using *in vitro* bioreactor culture system, we demonstrate that mechanical loading directly modulates the spatial localization and structure of collagens II and X. Furthermore, we show that mechanical loading *in vitro* rescues aspects of the development of collagens II and X from the effects of fetal immobility. In conclusion, our findings show that mechanical loading is a critical determinant of collagen network establishment during prenatal skeletal development.

## 1. Introduction

The matrix of articular cartilage has an extraordinarily complex structure and composition, which enables it to sustain high loads and facilitate almost frictionless movements throughout a lifetime, while the extracellular matrix of bone facilitates the strength and toughness of the tissue. The tensile properties of cartilage and bone depend on an intricate collagen network comprising multiple types of individual collagens. Numerous syndromes and disorders in which specific collagens are abnormal impact on skeletal development [1], including osteogenesis imperfecta [2] and chondrodysplasias [3]. Changes in the collagen network also play a role in age-related degeneration and the aetiology of osteochondral disorders, including osteoarthritis [4].

Articular cartilage and bone tissues arise from a cartilage template which forms in the early embryo [5]. As prenatal development of a long bone progresses, most of the cartilage is replaced through endochondral ossification. While articular cartilage was previously believed to form appositionally from the cartilage template [6,7], recent work has demonstrated that articular cartilage develops mainly interstitially [8]. Therefore, while the matrix of the skeletal tissues is substantially remodelled over ontogeny, the origins of the mature articular cartilage and bone matrix lie in prenatal development. In prior work, we revealed the dynamic changes in collagen fibre organization and complexity that occur over prenatal skeletal development, particularly in collagens II, X and XI [9]. The dynamism of collagen network emergence is likely to be critical to the functional and biological properties of the mature skeletal tissues, but the mechanisms underlying the increasing complexity of the multiscale collagen network over development are unknown.

Mechanical loading plays a critical role in the maintenance and health of mature and ageing skeletal tissues [10]. Mechanical loading is also critical to prenatal and postnatal skeletal development. In humans, a range of conditions in which fetal movement is reduced, restricted or (in extreme cases) absent have provided clinical evidence for the importance of muscle contractions for normal limb development. Decreased fetal movement is implicated in temporary brittle bone disease in infants [11] and fetal akinesia deformation sequence where new born present with limb deformities and abnormal joint contractures [12,13]. Neuromuscular disorders such as spinal muscular atrophy [14] and myotonic dystrophy [15] can lead to smaller, thinner and weaker bones, prone to postnatal fracture [16,17]. In our recent studies, we have reported that when skeletal muscle is absent or non-contractile in animal models, skeletal joint morphology is abnormal, joints tend to be fused, rudiment elongation is impaired and rudiment mineralization along with chondrocyte volume is reduced (they recover later in development) [18], and various aspects of vertebral structure such as segmentation, shape and intervertebral disc formation are all impacted [19,20]. While skeletal muscle contractions are crucial for normal growth and morphogenesis of most bones in the limbs and spine [18,21–28], we are not aware of prior studies which describe the effects of abnormal fetal movements on the extracellular matrix of skeletal tissues, or in particular, the temporal effects of mechanical forces on the extracellular matrix emergence over skeletal development.

It has been postulated that mechanical loading due to physical activity regulates the emergence of complexity in the collagen network of postnatal articular cartilage [5,29,30]. Mechanical loading also influences the collagen network at the cellular level; tensile forces between collagen fibre bundles and the cell membrane protrusions (fibripositors) from which collagen fibrils exit the cell have been proposed as drivers of collagen fibril formation, orientation and organization [31]. In this study, we hypothesize that mechanical loading is a critical factor in the initiation and maturation of the collagen network during prenatal skeletogenesis. To test this hypothesis, we first studied the effects of skeletal muscle contractions on the localization and structure of key skeletal collagens during prenatal development, using the Splotch-delayed mouse model which has no skeletal muscles (Pax3^spd−/−^, or ‘muscleless-limb’). Next, we determined if the effects of absent muscle loading on the collagen network are mediated directly by changes in mechanical loading, rather than by changes in the biochemical environment when skeletal muscle is missing. Fetal limb explants were cultured under static or dynamically loaded conditions in a mechano-stimulation bioreactor, to assess the impact of mechanical loading on collagen network development independently of muscle contractions. Finally, we tested if *in vitro* application of mechanical loading could ‘rescue’ the effects of fetal immobility on collagen network development. Understanding the contribution of mechanical loading to collagen network establishment and maturation provides insight into skeletal development and disease, and has the potential to lead to therapies for babies and children affected by reduced or abnormal movements.

## 2. Results

### 2.1. Collagens I, II, V, VI, X and XI are abnormal in the developing rudiment when muscle is absent

In order to investigate the role of skeletal muscle contractions *in utero* on prenatal skeletal development, we used a mouse line in which no limb skeletal muscles form in embryos homozygous for the mutation; Pax3^spd−/−^, or Splotch-delayed, referred to herein as ‘muscleless-limb’ mice. Three significant developmental stages of rudiment development were studied: Theiler stage (TS)22 [32] (typically embryonic day (e)13.5), the latest stage at which the rudiment is fully cartilaginous, TS25 (typically e16.5) when the growth plate and mineralized regions are present and TS27 (typically e18.5) the latest prenatal stage that can be reliably analysed. Muscleless-limb embryos and their normal littermates were harvested and staged. Seven collagen types (collagens I–III, V, VI, X and XI) were studied with immunofluorescence on cryosections of the humerus and imaged with confocal microscopy. The selected seven collagens were the same as those we characterized for normal development, except for collagen IX, which was not included in the current study due to the lack of any observed changes over normal development [9]. Confocal images were processed using FIJI [33] and qualitatively analysed to identify differences in the spatial localization between wild-type and muscleless-limbs over the developmental time course.

The absence of skeletal muscle did not lead to any obvious changes in collagen III at any of the stages studied (electronic supplementary material, figure S3). All other collagens studied were affected by the lack of skeletal muscle in terms of their structural organizations and/or their spatial localization, as described in detail below. Results shown are representative of all repeats unless otherwise described, and the complete dataset is provided in electronic supplementary material, figures S1–S8.

**Figure 1.**
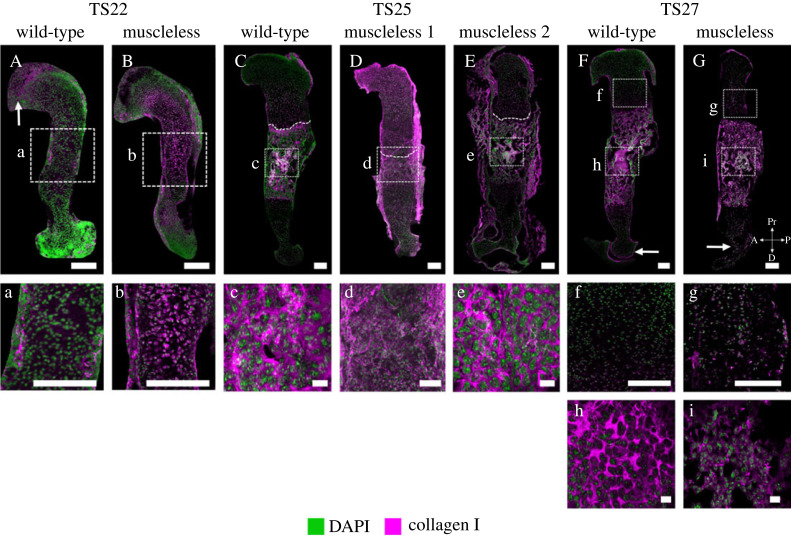
Both structure and localization of collagen I were abnormal in muscleless-limbs, with the effects varying according to the developmental stage. (A,B, a,b) In the TS22 wild-types, collagen I localization was concentrated in the perichondrium (A, a), and at the proximal epiphysis (A, arrow). In the muscleless-limbs at TS22 (B, b), there was localization of collagen I throughout much of the diaphysis, lacking specificity to the perichondrium. (C–E, c–e) At TS25, collagen I localization in the wild-types was concentrated in the mineralizing region and in the perichondrium (C). One of the muscleless-limbs at TS25 (Muscleless 1) had abnormal collagen localization throughout the cartilaginous region (D), lacked a clear cartilage to mineralization interface (D, dashed line), and also lacked the distinct collagen I fibre organization (d) seen in the wild-type rudiments (C, c). The other TS25 sample ‘Muscleless 2’ (E, e) had similar collagen I localization and organization to the wild-type. Dashed lines in (C–E) demarcate mineralizing and non-mineralized regions. (F,G, f,g) At TS27, both structure and localization of collagen I were abnormal when skeletal muscles were absent. Muscleless-limbs lacked collagen I localization in the distal perichondrium (compare arrows in F and G). Collagen I was seen throughout the non-mineralized cartilage in the muscleless-limbs (g) but not in the wild-types (f). In the mineralizing region, collagen I fibres in the muscleless-limbs were finer and less interconnected than those of wild-types (h,i). Except for the scale bars in (c), (e), (h) and (i), which show 10 µm, all scale bars are 200 µm. Sample numbers: TS22 wild-type/muscleless (*n* = 3), TS25 wild-type (*n* = 3), TS25 muscleless (*n* = 2), TS27 wild-type (*n* = 4), TS27 muscleless (*n* = 3).

*Collagen I* is normally localized to the perichondrium and the mineralizing cartilage and plays an important role in bone mass and strength [34]. In the TS22 wild-types, collagen I localization was concentrated in the perichondrium (figure 1A, a), and at the proximal epiphysis (figure 1A, arrow). In the muscleless-limbs at TS22, there was localization of collagen I throughout much of the diaphysis, lacking specificity to the perichondrium (figure 1B, b). At TS25 and TS27, collagen I localization in the wild-types was concentrated in the mineralizing region and in the perichondrium (figure 1C,F). One of the two muscleless-limbs studied at TS25 (Muscleless 1) exhibited diffuse collagen localization throughout the cartilaginous region (figure 1D), and did not exhibit a clear cartilage to mineralization interface (figure 1D, dashed line) or the distinct collagen I fibre organization (figure 1D) seen in the wild-type rudiments (figure 1C, c). Collagen I localization and organization in the other TS25 muscleless-limb (‘Muscleless 2’; figure 1E, e) were similar to the TS25 wild-types (figure 1C, c). At TS27, both structure and localization of collagen I were abnormal when skeletal muscles were absent. TS27 muscleless-limbs lacked collagen I localization in the distal perichondrium (compare arrows in figure 1F,G). Collagen I was seen throughout the non-mineralized cartilage in the TS27 muscleless-limbs (figure 1g) but not in the wild-types (figure 1f). In the mineralizing region, the collagen I fibres in the muscleless-limbs appeared finer and less connected than those of wild-types (figure 1h,i). Therefore, when skeletal muscles were lacking, the structure and localization of collagen I was aberrant, with the consequences changing with developmental stage.

**Figure 2.**
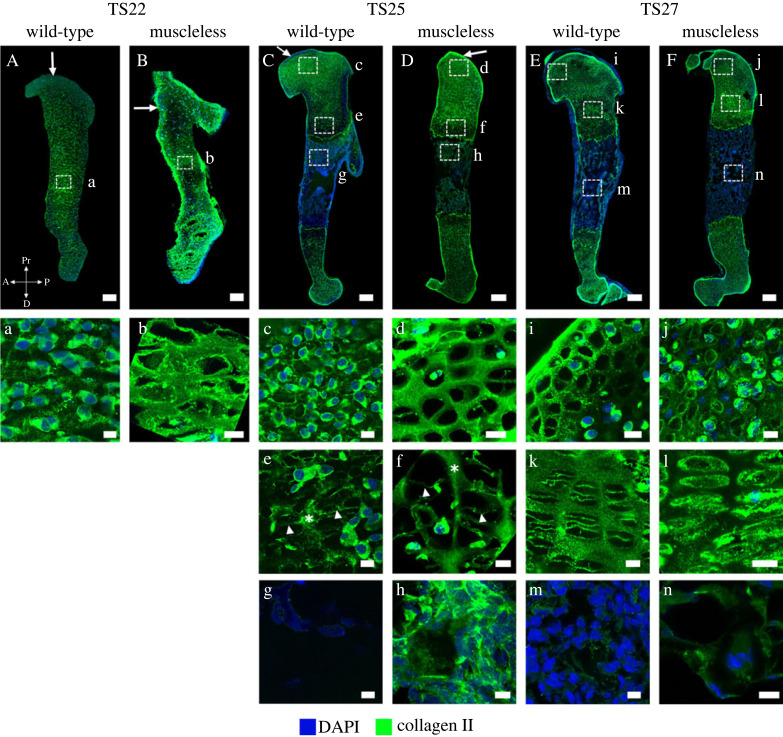
Collagen II exhibited an initially more structured organization in muscleless-limbs than in wild-type limbs, but this structure was lost by TS27, when the wild-type characteristic meshwork pattern was fully established. (A,B, a,b) At TS22, muscleless-limbs exhibited widespread perichondral localization (including the future articular cartilage region), whereas wild-type limbs had only mild collagen II staining in the perichondrium at this stage (arrows in A,B). A meshwork-like pattern was already evident in the mid-diaphysis of muscleless-limbs, while no such pattern was obvious in the wild-types (a, b). This well organized architecture of collagen II in the muscleless-limbs concealed chondrocyte nuclei leading to appearance of low dapi staining in these sections. (C,D, c–h) At TS25, perichondral localization of collagen II in the muscleless-limbs was more pronounced than in the wild-type limbs (arrows in C,D). Muscleless-limbs exhibited a prominent collagen II meshwork in the proximal humerus with thick interconnected bundles (d), not observed in the wild-types (c). The hypertrophic regions of the muscleless-limbs had thick bundles of collagen II localization in the longitudinal septa (f, star) with thinner bundles in the transverse septa (f, arrowhead), a pattern that was more pronounced compared to that seen in the wild-types (e). The sparely distributed DAPI staining in these sections was due to large size of hypertrophic chondrocytes. In the TS25 mineralizing regions, muscleless-limbs had a fibrous collagen II organization (h), whereas no collagen II was present in the mineralizing regions in the wild-type rudiments (g). (E,F, i–n) By TS27, no differences in collagen II localization between muscleless-limbs and wild-types were evident (E,F). The characteristic meshwork pattern of collagen II in the proximal humerus was almost completely lost in the muscleless-limbs, but fully established in the wild-types (i,j). A distinctive septal organization was established throughout the growth plates of the TS27 wild-types (k) while no longer present in the muscleless-limbs (l). Mild collagen II immunopositivity was observed in the mineralized region of the muscleless-limbs (n) but not in the wild-types (m). Scale bars, 88 µm (A,B), 200 µm (C–G) and 10 µm (a–n). Sample numbers: TS22 wild-type (*n* = 4), TS22 muscleless (*n* = 3), TS25 wild-type (*n* = 3), TS25 muscleless (*n* = 2), TS27 wild-type/muscleless (*n* = 3).

*Collagen II* is the major collagen of cartilage, normally present in the non-mineralized regions of cartilaginous rudiments and, later in prenatal development, the perichondrium [9]. Collagen II is necessary for the proper formation of articular cartilage, the epiphyseal growth plate, endochondral bone and the intervertebral disc [35,36]. Both spatial distribution and structural organization of collagen II were affected by the lack of skeletal muscles. At TS22, the muscleless-limbs exhibited widespread perichondral localization (including the future articular cartilage region), whereas wild-type limbs had only mild collagen II staining in the perichondrium at this stage (figure 2A,B, arrows). A meshwork-like pattern was already evident in the muscleless-limbs, while no such pattern was obvious in the wild-types (figure 2a,b). At TS25, perichondral localization of collagen II in the muscleless-limbs was much more pronounced and intense than in the wild-type limbs (figure 2C,D, arrows). TS25 muscleless-limbs exhibited a prominent collagen II meshwork in the proximal humerus with thick interconnected bundles (figure 2d), which was not observed in the wild-types (figure 2c). The hypertrophic regions of the muscleless rudiments had thick bundles of collagen II localization in the longitudinal septa (figure 2f, star) with thinner bundles in the transverse septa (figure 2f, arrowhead), a pattern that was much more pronounced compared to that seen in the wild-types (figure 2e, star and arrowhead). In the TS25 mineralized regions, muscleless-limbs had a fibrous collagen II organization (figure 2h) whereas no collagen II was present in the mineralized regions in the wild-type rudiments (figure 2g). By TS27, no differences in collagen II localization between muscleless-limbs and wild-types were evident (figure 2E,F). However, the characteristic meshwork pattern of collagen II in the proximal humerus was almost completely lost in the muscleless-limbs, but fully established in the wild-types (figure 2i,j). A distinctive septal organization was established throughout the growth plates of the TS27 wild-types (figure 2k) while no longer present in the muscleless-limbs (figure 2l). Mild collagen II immunopositivity was observed in the mineralized region of the muscleless-limbs (figure 2n) but not in the wild-types (figure 2m). Therefore, early in development, the absence of skeletal muscle led to abnormal collagen II localization in the perichondrium, and a precocious structural arrangement in the epiphyseal cartilage. However, by TS27, the muscleless-limbs had lost all of the structural arrangement of collagen II, the same stage at which the wild-type limbs have a pronounced structure.

**Figure 3.**
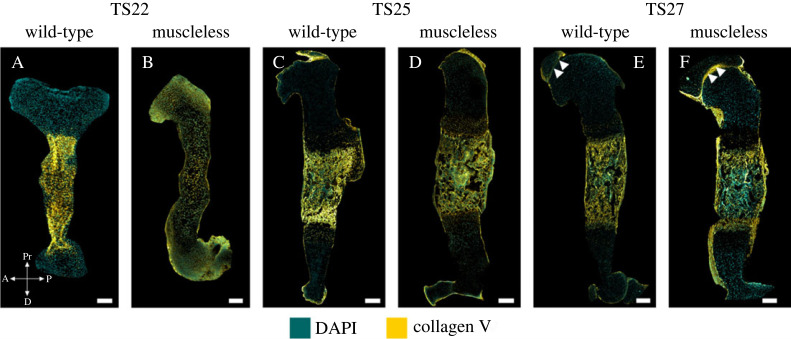
Collagen V localization was aberrant at TS22 and TS27 in muscleless-limbs, with no evident structural abnormalities. (A,B) At TS22, collagen V was present throughout the muscleless-limbs (B), whereas there was consistent diaphyseal localization in the wild-type rudiments, without any epiphyseal localization (A). (C,D) At TS25, there were no pronounced abnormalities in localization of collagen V. (E,F) At TS27, the muscleless-limbs had more pronounced collagen V localization in the perichondrium and the proximal joint region compared to the wild-types (arrowheads in E,F). Scale bars, 200 µm. Sample numbers: TS22 wild-type/muscleless (*n* = 3), TS25 wild-type (*n* = 3), TS25 muscleless (*n* *=* 2), TS27 wild-type/muscleless (*n* = 3).

*Collagen V* is important for the regulation of collagen I fibre number and diameter [37–40] and therefore plays an important role in bone development. Several aspects of collagen V localization were abnormal in the muscleless-limbs, while no pronounced abnormalities in structure were evident at any stage (figure 3). At TS22, collagen V was present throughout the muscleless-limbs (figure 3B), whereas there was consistent diaphyseal localization in the wild-type rudiments, without any epiphyseal localization (figure 3A). At TS25, there were no pronounced abnormalities in localization of collagen V (figure 3C,D). However, at TS27, the muscleless-limbs had more pronounced collagen V localization in the perichondrium and the proximal joint region (figure 3F, arrowheads) compared to the wild-types (figure 3E, arrowheads). Therefore, the absence of muscles led to abnormal localization of collagen V at two of the three stages examined, with no obvious effects on structure.

**Figure 4.**
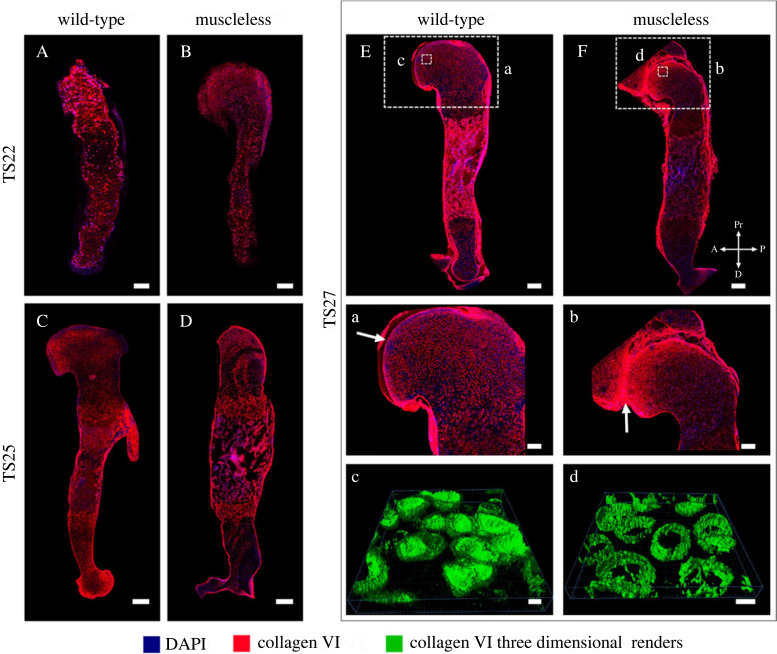
Effects of absent skeletal muscle on collagen VI were only detected at the last stage studied, with increased joint line localization and altered chondron morphology in the TS27 muscleless-limbs. (A–D) The overall spatial distribution of collagen VI was not visibly affected by the lack of skeletal muscle at TS22 (a*,*b) or at TS25 (C,D). (E,F, a–d) At TS27, there was a pronounced increase in staining intensity of collagen VI in the proximal humerus in the muscleless-limbs compared to the tight joint line localization in the wild-types at the same stage (a,b, arrows). While the TS27 chondrons had an ovoid convexity in the wild-types (c), the muscleless-limb chondrons at the same stage were more cylindrical in shape (d). Scale bars, 200 µm (A–F), 100 µm (a,b) and 5 µm (c,d). Sample numbers: TS22 wild-type/muscleless (*n* = 3), TS25 wild-type (*n* = 3), TS25 muscleless (*n* = 2), TS27 wild-type/muscleless (*n* = 3).

*Collagen VI* is localized to the pericellular matrix and delineates chondron structure [41]. The pericellular matrix is a key player in chondrocyte mechanotransduction [42]. While the overall spatial distribution of collagen VI was not visibly affected by the lack of skeletal muscle at TS22 and TS25 (figure 4A–D), there was a pronounced increase in staining intensity of collagen VI in the proximal humerus in the muscleless-limbs at TS27 compared to the tight joint line localization in the wild-types at the same stage (figure 4a,b, arrows). Chondron shapes appeared normal at TS22 and TS25 (electronic supplementary material, figure S5), but structural differences in chondron shape were observed in the proximal humerus by TS27. While the TS27 chondrons had an ovoid convexity in the wild-types (figure 4c), the muscleless-limb chondrons at the same stage were more cylindrical in shape (figure 4d). Therefore, a lack of skeletal muscle affected collagen VI only at latest stage examined, with increased joint line localization and altered chondron shape.

**Figure 5.**
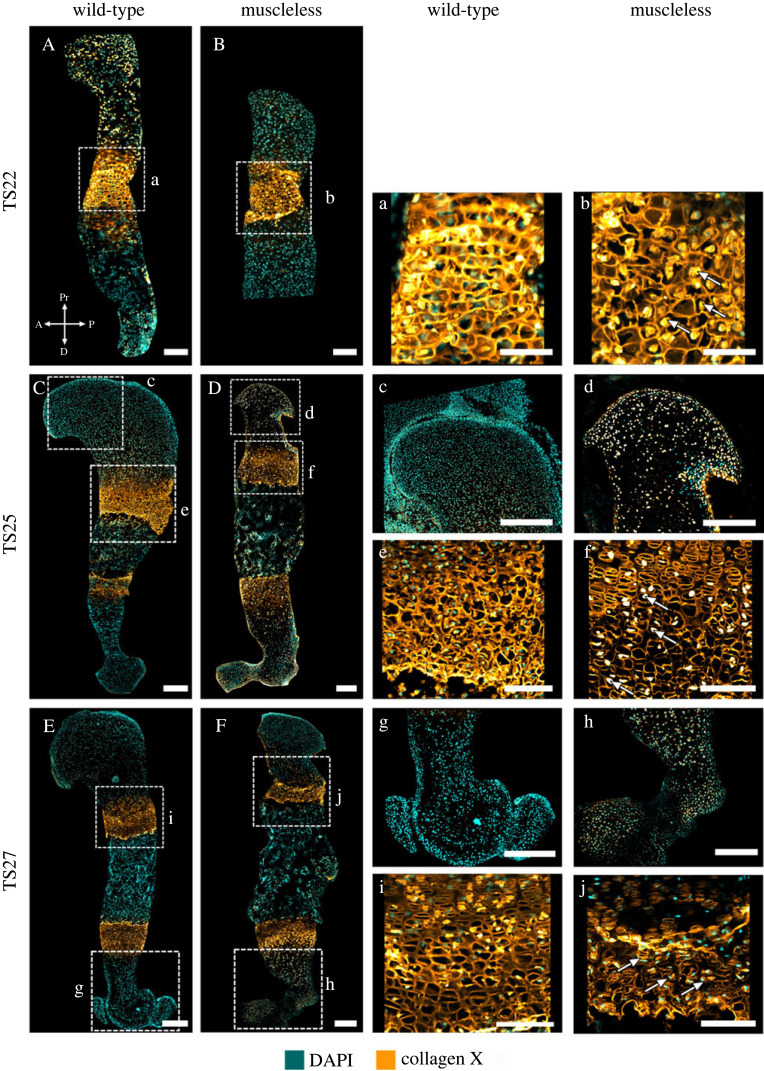
At all three stages studied, muscleless-limbs had increased intracellular localization of collagen X, and aberrant growth plate structure. (A,B, a,b) The wild-type collagen X structure at TS22 had an intricate weave with an obvious anterior–posterior grid-like pattern (a), whereas the structure was more randomly oriented in the muscleless-limbs (b). The muscleless-limbs had increased intracellular localization in the growth plates (b, arrows). (C,D, c–f) At TS25, collagen X was strongly localized to the mineralizing cartilage in wild-types (C, c), while staining in the non-mineralized cartilage was widespread in the muscleless-limbs (D, d). As at TS22, the TS25 muscleless-limbs had increased intracellular localization in the growth plates (f, arrows). There was a less defined interlocking arrangement of collagen X in the muscleless-limb growth plate (f) than in the wild-type growth plate (e). (E,F, g–j) At TS27, staining in the non-mineralized cartilage was still more pronounced and widespread in the muscleless-limbs (F, h) than in the wild-types (E, g). TS27 muscleless-limbs had increased intracellular localization in the growth plates (j, arrows). In the wild-type limbs, collagen X had a columnar arrangement in the pre-hypertrophic region, and an oblique, orientation in the hypertrophic regions (i). In the muscleless-limbs, there was no complexity of structure within the different parts of the growth plate, with only an approximate columnar arrangement (j). Scale bars, 200 µm (A–F), 65 µm (a–b), 170 µm (c) and 100 µm (d–j). Sample numbers: TS22 wild-type (*n* = 3), TS22 muscleless (*n* = 2), TS25 wild-type (*n* = 3), TS25 muscleless (*n* = 2), TS27 wild-type/muscleless (*n* = 3).

*Collagen X* is an important component of the growth plate [43] and, during development, is normally present in the pre-hypertrophic and hypertrophic growth plate regions [9]. Both localization and structure of collagen X were affected by the absence of skeletal muscle. In wild-type limbs, collagen X was strongly localized to the mineralizing cartilage, with some sparse staining in the non-mineralized cartilage (figure 5A,C,E, c,g). This staining in the non-mineralized cartilage was much more widespread and pronounced in the muscleless-limbs at TS25 and TS27 than in the wild-types (compare figure 5d,c, and h,g). In the growth plates of the wild-type limbs, collagen X staining was mainly extracellular (figure 5a,e,i), while in the muscleless-limbs, there was a tendency across all three stages for increased, intracellular localization in the growth plates, as indicated with arrows in figure 5b,f,j. The structure of collagen X was also affected by the lack of skeletal muscle. At TS22, both wild-type and muscleless-limbs had a hexagonal collagen X structure in the mid-diaphysis (figure 5a,b). However, the wild-type collagen X structure at TS22 had an intricate weave with an obvious anterior-posterior grid-like pattern (figure 5a), whereas the structure was more randomly oriented in the muscleless-limbs at the same stage (figure 5b). In the wild-types at TS25, collagen X exhibited a complex interlocking arrangement in the growth plate (figure 5e), while once again the organization in the muscleless-limbs was less structured (figure 5f). In the TS27 wild-type limbs, collagen X had a columnar arrangement in the pre-hypertrophic region, and a teardrop, or oblique, orientation in the hypertrophic regions (figure 5i). By contrast, in the muscleless-limb rudiments at TS27, there was no complexity of structure within the different parts of the growth plate, with only an approximate columnar arrangement (figure 5j). Therefore, the absence of skeletal muscle led to increased staining of collagen X in the non-mineralized cartilage, increased intracellular localization in the mineralizing cartilage, and pronounced effects on the structure of collagen X in the growth plate at all three stages examined.

**Figure 6.**
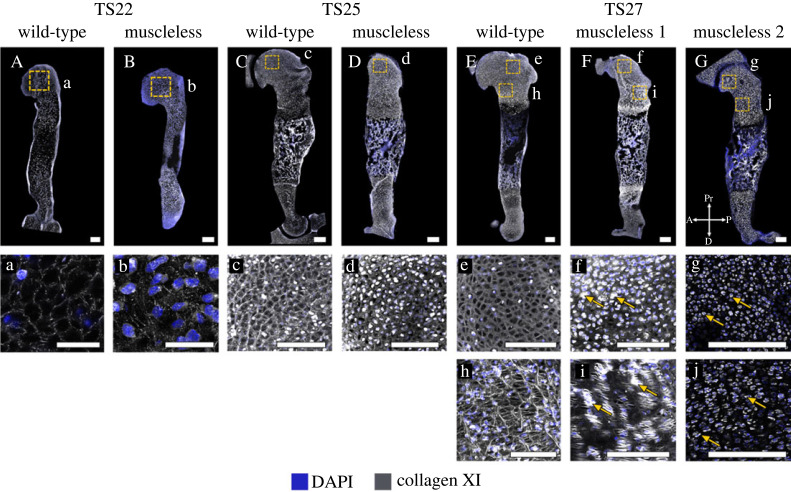
Effects of absent skeletal muscle on collagen XI were only detected at the last stage studied, with substantially altered structure in the TS27 muscleless-limbs. (A–D, a–d) No substantial differences in collagen XI structure or localization were observed between wild-type and the muscleless-limbs at TS22 (Aa, Bb) or TS25 (Cc, Dd). (E–G, e–j) At TS27, dramatic differences in collagen XI were observed between the wild-type and the muscleless-limbs in all regions. In two out of the three samples, muscleless-limbs had no meshwork pattern in the proximal humerus and no distinct septal organization in the growth plate regions (E,F, h,i). In the third muscleless-limb sample, there was a complete absence of any collagen XI extracellular matrix arrangement (G, g,j). TS27 muscleless-limb rudiments also had increased intracellular collagen localization compared to wild-types (arrows, f,g,i,j). Scale bars, 200 µm. Sample numbers: TS22 wild-type (*n* = 3), TS22 muscleless (*n* = 2), TS25 wild-type (*n* = 3), TS25 muscleless (*n* = 1), TS27 wild-type/muscleless (*n* = 3).

*Collagen XI* is important for chondrogenesis and has a protective role in maintaining healthy cartilage [44]. It co-polymerizes with collagen II [45] and, during development, is predominantly present in non-mineralized cartilage [9]. No substantial differences in collagen XI structure or localization were observed between wild-type and the muscleless-limbs at TS22 (figure 6A,B, a,b) or TS25 (figure 6C,D, c,d). However, by TS27, dramatic differences were observed between the wild-type and the muscleless-limbs in all regions. In two out of the three samples, muscleless-limbs lacked the meshwork pattern in the proximal humerus and also lacked a distinct septal organization in the growth plate regions (figure 6E,F, e,f,h,i). In the third muscleless-limb sample, there was a complete absence of any collagen XI extracellular matrix arrangement (figure 6G, g,j). Also at TS27, muscleless-limb rudiments had increased intracellular collagen localization compared to wild-types (figure 6f,g,i,j, arrows). Therefore, the structure and localization of collagen XI initiated apparently normally when skeletal muscle was absent, but the structure of collagen XI was dramatically affected by the lack of skeletal muscle by TS27, as was the balance between extracellular and intracellular localization.

### 2.2. Mechanostimulation bioreactor culture of wild-type and muscleless-limb rudiments

**Figure 7.**
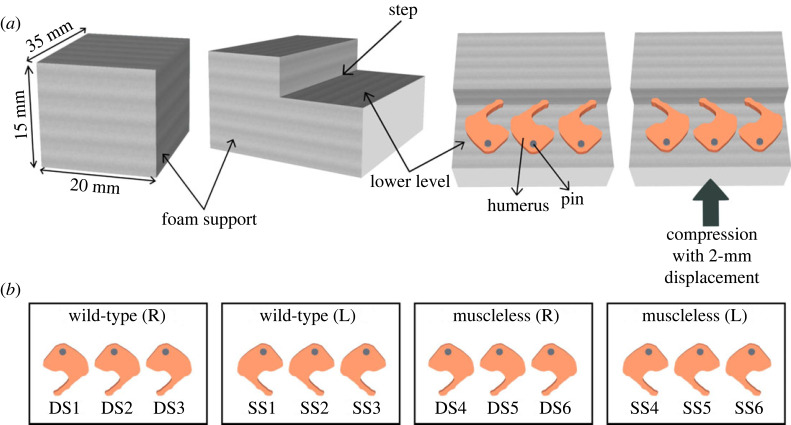
Schematic of the experimental set-up for the mechanostimulation of wild-type and muscleless humeri. (*a*) Three limbs pinned to foam supports were placed in the bioreactor chamber for dynamic culture or Petri dishes for static culture. The loading regime was comprised of cyclical compression of the forelimbs at 0.67 Hz to a displacement of 2 mm for 2 h, three times per day. This produced approximately 14° angle cyclic flexion of the elbow [46]. (*b*) Schematic of the comparison groups to highlight that the contralateral humerus from the same embryo served as paired samples for dynamic and static groups.

Our immunofluorescence data demonstrates that the establishment of a normal collagen network is dependent on skeletal muscles. The impact of the muscles could be due to (i) mechanical loading, (ii) biochemical signalling from the muscles or (iii) a combination of the two. To test the hypothesis that the change in mechanical loading is the dominant factor affecting collagen organization when skeletal muscle is absent, we performed bioreactor experiments in which limb explants underwent either *in vitro* dynamic mechanical stimulation or static culture (figure 7). Embryos were harvested at e15.5, which, in our hands, yields embryos at around TS24. From each embryo, one forelimb was cultured under dynamic loading conditions and the contralateral limb under static conditions, over a culture period of 6 days. Limbs were loaded three times per day, for 2 h each time, under cyclical compression at 0.67 Hz. An applied displacement of 2 mm induced a flexion of approximately 14° at the elbow. Paired comparisons of the collagens most dramatically affected by the absence of muscles *in vivo* (collagens II, X and XI) were made between dynamically cultured and contralateral statically cultured forelimbs.

### 2.3. Dynamic loading *in vitro* affects localization and structure of collagens II and X, but not XI

**Figure 8.**
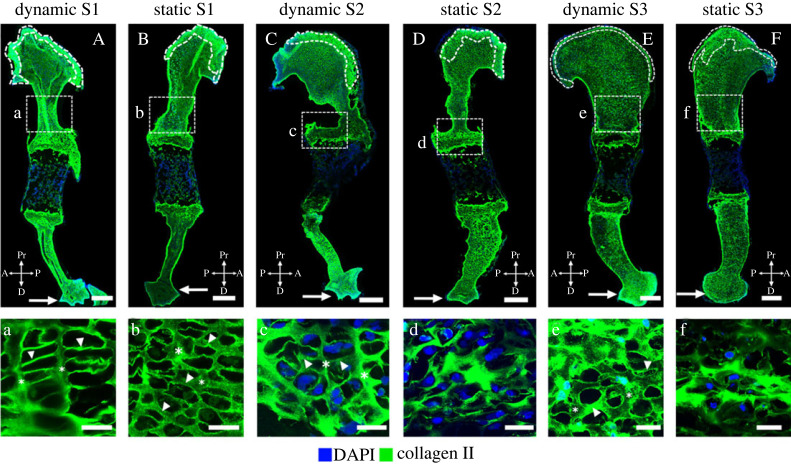
Collagen II localization and structure were regulated by *in vitro* mechanical loading in wild-type explants. (A–F) Dynamic loading promoted localization of collagen II in the proximal future articular cartilage regions and in the distal epiphysis; compare the distinct, uniform band of collagen II distribution in the future articular cartilage region of dynamically stimulated limbs (dashed lines in A,C,E) with the absence of a distinct boundary between the band of collagen II and the rest of the humeral head in contralateral statically cultured limbs (dashed lines in B,D,F). Collagen II immunopositivity was stronger in the distal humeri of dynamically stimulated limbs than in the statically cultured contralateral limbs (arrows in A–F). Dynamic loading *in vitro* also led to a more structured organization of collagen II than seen in statically cultured contralateral limbs; compare the longitudinal septa (*) and transverse septa (arrowheads) between a and b, c and d, and e and f. Scale bars, 200 µm (A–F), 20 µm (a–e). Sample numbers: *n* = 3 embryos, right limbs cultured under dynamic loading and left limbs cultured under static conditions.

Dynamic loading promoted localization of collagen II in the proximal future articular cartilage regions and in the distal epiphysis. In the dynamically cultured limbs, we consistently observed a distinct, uniform band of collagen II distribution in the future articular cartilage region of the humeral head (figure 8A,C,E, dashed lines), as also seen *in vivo* at TS27 (figure 2). In the proximal humerus of statically cultured limbs, collagen II localization extended beyond the future articular cartilage region into the epiphysis (figure 8B,D,F, dashed lines), leading to the loss of a distinct boundary between the future articular cartilage region and the rest of the humeral head. Dynamically cultured limbs had a stronger collagen II immunopositivity in the distal humerus compared to the statically cultured explants (figure 8A–F, arrows). Dynamic culture also led to differences in collagen II structure in the proliferative region of the growth plate, but there was variation between the three samples analysed, as shown in figure 8. In the dynamically cultured limbs of samples one and two (S1, S2), the collagen II organization was similar to that normally seen at TS27 (figure 2), with distinct longitudinal (figure 7a,c, stars) and transverse septal organization (figure 8a,c, arrowheads). Localization was more pronounced in the longitudinal septa than in the transverse septa, leading to a ‘rungs on a ladder-like’ organization. In the statically cultured contralateral limbs of the same samples, a clear structure was present (figure 8B*,*D), but there were no differences in collagen organization between the longitudinal (figure 8b,d, stars) and transverse septa (figure 8b,d, arrowheads). In the third sample (S3), dynamic loading led to a structure which was not as pronounced as that of the other two dynamically cultured limbs (figure 8e), and yet a structured arrangement was still present with a prominent transverse septa (figure 8e, arrowheads) and mild localization in the longitudinal septa (figure 8e, stars). By contrast, no septal organization was observed in the cartilage of the statically cultured contralateral limb of S3, where collagen II was present throughout the matrix with large cell-shaped voids (figure 8f). The variation in effects of loading on collagen II structure between S1/S2 and S3 is likely due to differences in the developmental stage of the animals, with sample three being more developmentally mature than the other two (as indicated by more pronounced morphology of the proximal and distal humerus in sample three; electronic supplementary material, figure S9). These findings demonstrate the importance of mechanical loading for collagen II localization and structure, and also suggest that the effects of mechanical stimulation on collagen II organization depend on the rudiment developmental stage.

**Figure 9.**
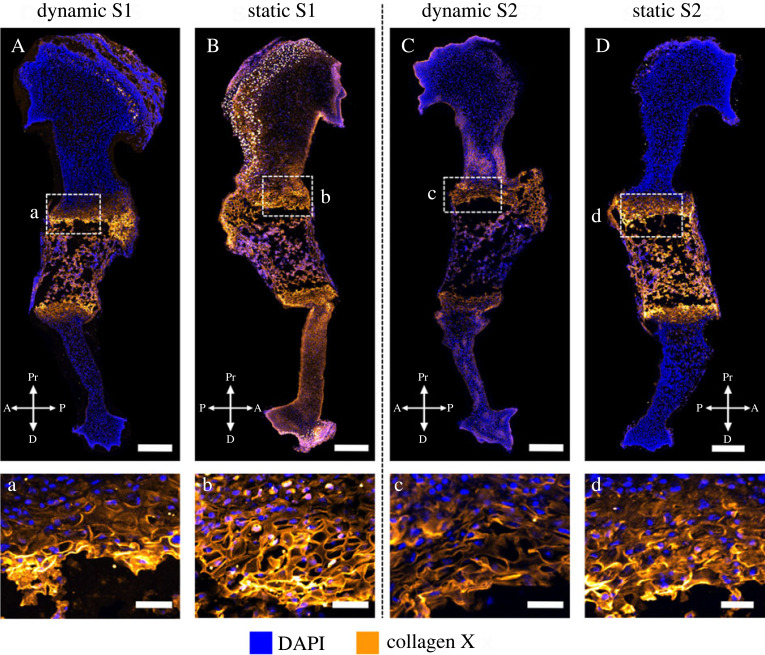
External mechanical loading determined localization of collagen X in wild-type explants. (A–D) Dynamic loading *in vitro* led to more normal localization of collagen X than static culture, with statically cultured limbs having more extensive collagen X in the non-mineralized cartilage (B) or in the mid-diaphysis (D). (a–d) Dynamic loading *in vitro* had no obvious effects on the collagen X structure in the growth plate. Scale bars, 100 µm (A–D) and 20 µm (a–d). Sample numbers: *n* = 2 embryos, right limbs cultured under dynamic loading, left limbs cultured under static conditions.

Dynamic loading *in vitro* led to more normal localization of collagen X than static culture. As with collagen II, there was variation in the effects on collagen X between the limbs analysed, as shown in figure 9. In the set of forelimbs from sample one (S1), dynamic loading led to localization of collagen X to the growth plates (figure 9A) and mineralizing region (figure 9A), while the statically cultured contralateral rudiment had extensive collagen X in the non-mineralized cartilage (figure 9B). Therefore, while S1 did not have normal localization in the dynamically cultured limb (due to immunopositivity in the mineralized cartilage), the localization pattern was more normal than in the statically cultured limb. The strong immunopositivity observed in the non-mineralized cartilage of the statically cultured limb was characteristic of the muscleless-limb profile of collagen X at TS25 and TS27 (figure 5). In the second pair of forelimbs (S2), after dynamic loading, collagen X was localized mainly to the growth plates (figure 9C) with only mild immunopositivity in the mineralizing cartilage (figure 9C), while the static contralateral limb had strong immunopositivity in both the growth plate (figure 9D) and the mineralizing cartilage (figure 9D). There were no obvious differences in collagen X structure in the growth plate in either sample (figure 9a–d), with dynamic and static samples exhibiting a similar membrane-like configuration in this region similar to those seen in TS25 wild-types (figure 5). These results identify a role of mechanical loading for correct localization of collagen X in the developing limb.

**Figure 10.**
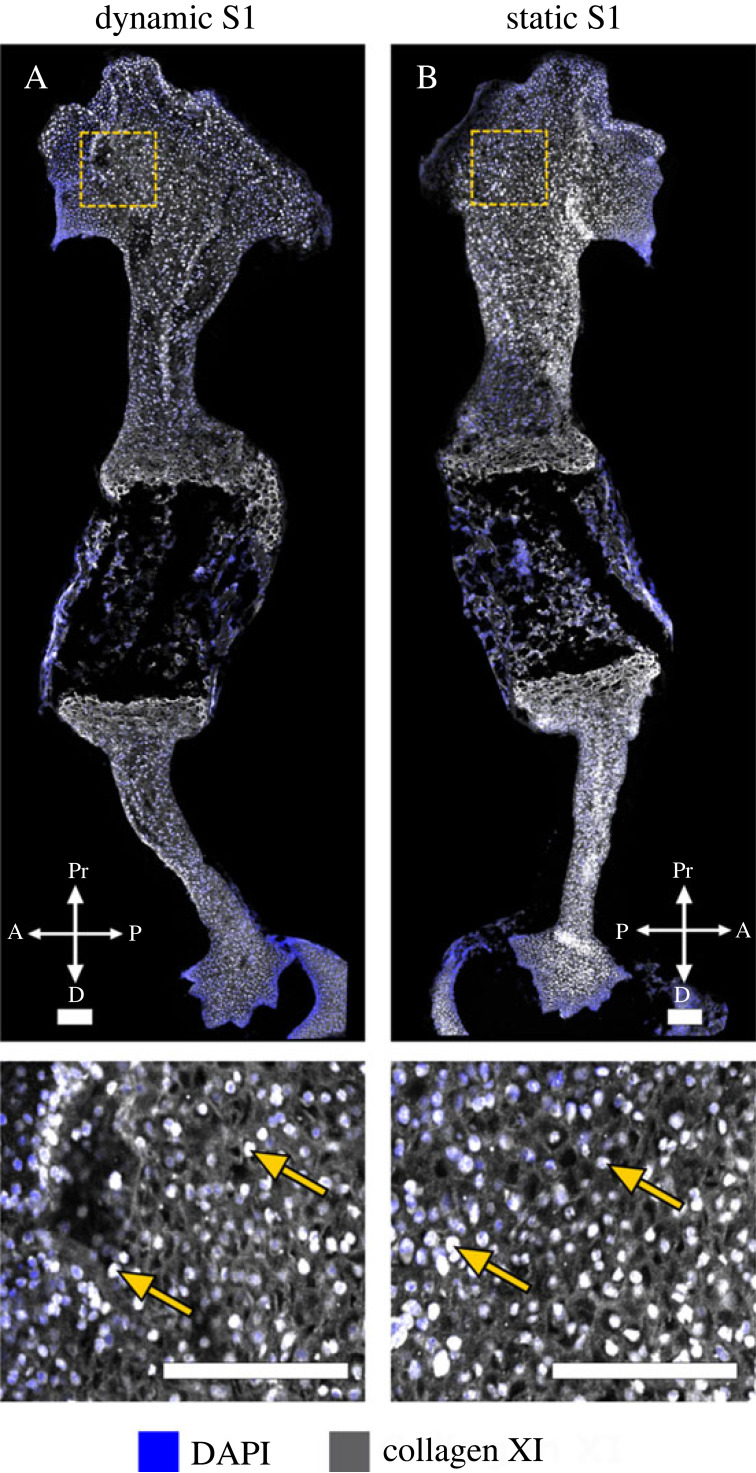
At the stage of development studied, external mechanical loading did not directly influence the structure or localization of collagen XI in wild-type explants. No difference in collagen XI organization was observed between dynamically (A) and statically (B) cultured limbs harvested at e15.5. There was widespread intracellular localization of collagen XI in both dynamically and statically cultured limbs (arrows). Scale bars, 100 µm. Sample numbers: *n* = 2 embryos, right limbs cultured under dynamic loading, left limbs cultured under static conditions.

Neither the structure nor the distribution of collagen XI were different between dynamic and static limbs in either of the two samples examined (figure 10). An indistinct mesh-like organization was observed throughout the rudiment in both dynamic and static limbs (figure 10). This pattern of localization and structure was not similar to any stages of the normal wild-type samples (figure 6). Prominent intracellular localization was observed in both sets of limbs (arrows in figure 10), as seen in the muscleless limbs at TS27 (figure 6). As collagen XI was not substantially affected by absent skeletal muscle *in vivo* until e18.5 (TS27; figure 6), it is possible that the rate of development and/or duration of culture were insufficient for effects of loading on the collagen XI network to become evident.

### 2.4. Dynamic mechanical loading *in vitro* rescues aspects of collagen network emergence in muscleless-limb explants

Our final experiment sought to investigate if *in vitro* mechanical loading could reverse or recover the adverse effects of fetal immobility on development of collagens II, X and XI, further corroborating a direct link between mechanical loading and the collagen network development, and identifying a possible therapeutic intervention for healthier skeletal development after a period of fetal akinesia. Muscleless-limbs were harvested at e15.5, or approximately TS24, at which stage collagens II and X were already abnormal in the mutants (figures 2 and 5), then cultured under static or dynamic conditions.

**Figure 11.**
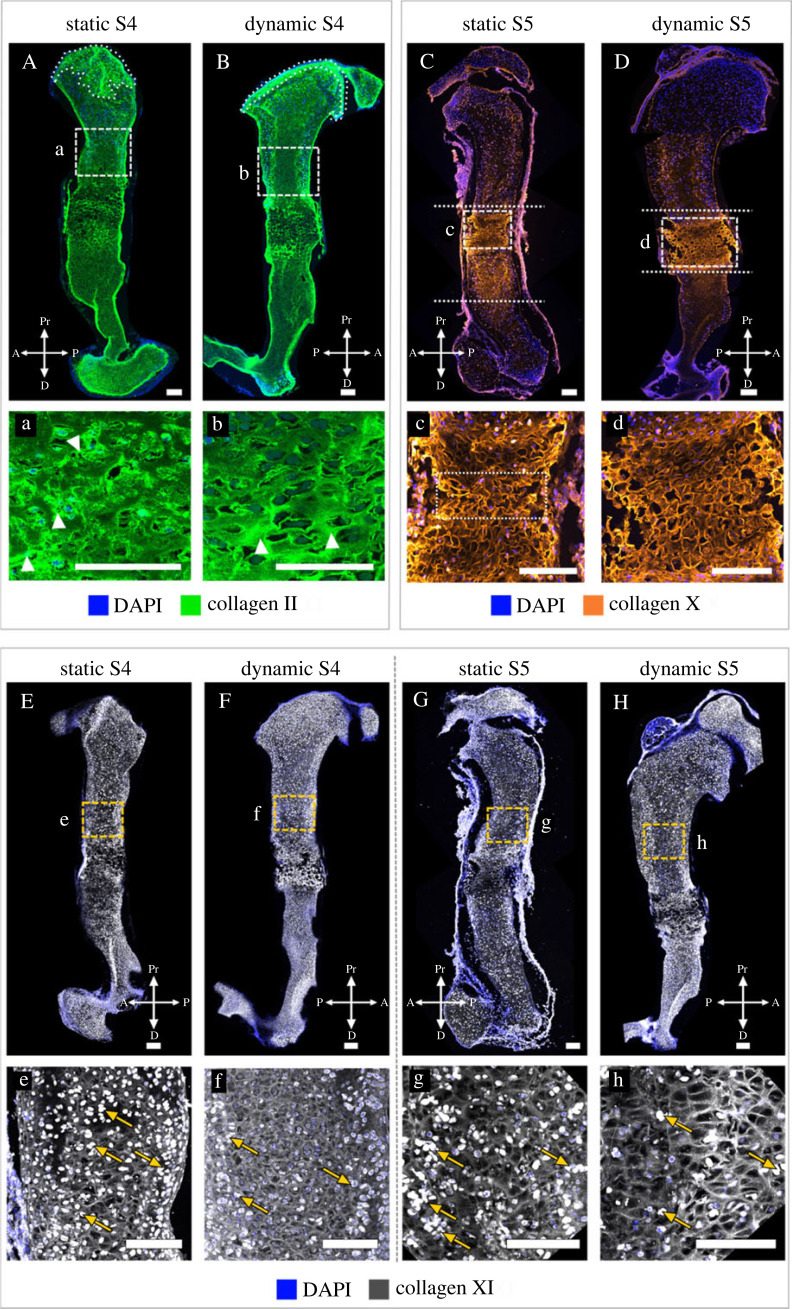
External mechanical loading partially restored the localization and structure of collagens II and X and had subtle effects on the localization and structure of collagen XI in cultured muscleless-limb explants. (A,B, a,b) *In vitro* loading of muscleless-limb explants restored more normal localization of collagen II in the future articular cartilage region (dotted areas in A,B) and rescued aspects of structure in the proliferative region of the growth plate, with distinct longitudinal and transverse septal arrangements evident in the dynamically cultured limbs (compare the longitudinal septa (arrowheads) and transverse septa between A and B). (C,D, c,d) *In vitro* loading of muscleless-limb explants promoted mid-diaphyseal localization of collagen X, with reduced and milder immunopositivity in the diaphysis and epiphyses (D) compared to the statically cultured contralateral limbs (C). Dynamic loading also promoted more normal collagen X structure in the muscleless-limb explants, with an intricate interlocking collagen X matrix organization without any collapsing regions in the loaded limbs (c,d). (E–H, e–h) Loading of muscleless-limbs *in vitro* led to a more pronounced collagen XI meshwork pattern (compare within contralateral limb pairs shown in E–H), and a reduction in intracellular localization compared to the statically cultured muscleless-limbs (arrows in e–h). Scale bars, 100 µm. Sample numbers: *n* = 3 embryos for collagens II and X, *n* = 2 for collagen XI, left limbs cultured under dynamic loading and right limbs cultured under static conditions.

Aberrant retention of collagen II in the mineralized region was seen in both statically and dynamically cultured muscleless-limbs (*n* = 3; figure 11A,B), indicating that abnormal localization of collagen II in muscleless-limbs at time of harvest could not be recovered by mechanical stimulation *in vitro*. Strong localization of collagen II to the future articular cartilage region was observed in the dynamically cultured, but not the statically cultured, muscleless-limbs (compare figure 11B and figure 11A, dotted lines), identifying a direct role of mechanical loading in specification of collagen II localization in the future articular cartilage region of the proximal humerus. With regards to the structure of collagen II (also aberrant in the muscleless-limbs at time of harvest), dynamic loading rescued aspects of structure in the proliferative region of the growth plate. In the dynamically cultured limbs, distinct longitudinal (figure 11b, arrowheads) and transverse septal (figure 11b) arrangements were evident, with the transverse septa surrounding cell-shaped voids in a columnar arrangement. The structure in the dynamically cultured limbs showed some resemblance to the structure seen in the TS25 wild-type rudiments (figure 2e). In the statically cultured contralateral limbs, no distinct longitudinal or transverse septal organizations of collagen II were observed (figure 11a). The thick bundles of collagen II in the static group lacked any obvious principle arrangement (figure 11a, arrowheads) and surrounded randomly oriented cell-shaped voids. Taken together, these data indicate that mechanical loading is critical to restricted localization of collagen II in the future articular cartilage region and to organization of collagen II in the proliferative region of the growth plate.

*In vitro* mechanical loading partially rescued the effects of fetal immobility on the localization and structure of collagen X in muscleless-limb explants. Intense collagen X staining was present outside of the mineralizing regions in both static and dynamic muscleless-limb explants (*n* = 3; figure 11C,D), as seen in muscleless-limbs *in vivo* and in statically cultured wild-type explants. However, dynamic loading *in vitro* promoted mid-diaphyseal localization of collagen X, with reduced and milder immunopositivity in the diaphysis and epiphyses (figure 11D) compared to the statically cultured contralateral limbs (figure 11C). Dynamic loading also promoted a more normal collagen X structure in the muscleless-limb explants. In the static group, collagen X had a membrane-like configuration with regions where the matrix had a collapsed appearance (figure 11c, dotted box). With dynamic loading, an intricate interlocking collagen X matrix organization without any collapsing regions was restored (figure 11d), somewhat resembling the oblique/teardrop appearance seen in the TS25 rudiments (figure 5e). Therefore, these data corroborate a direct link between mechanical loading and both localization and structure of collagen X.

As the effects of fetal immobility on collagen XI did not become evident until TS27 (figure 6), substantially after the time of harvest for culture (e15.5/TS24), the concept of rescue or recovery due to loading *in vitro* did therefore not apply for collagen XI. However, assessing the effects of external mechanical stimulation on collagen XI in the muscleless-limbs enabled us to test if the early establishment of the collagen XI network occurs completely independent of mechanical loading. Loading of muscleless-limbs *in vitro* led to a more pronounced collagen XI meshwork pattern (compare within contralateral limb pairs shown in figure 11E–H), and a reduction in intracellular localization compared to the statically cultured muscleless-limbs (arrows, figure 11e–h). These results are in contrast to the same experiment performed on limb explants with normal muscle, which showed no effects of *in vitro* loading on collagen XI (figure 10). Therefore, the influence of *in vitro* mechanical loading on the collagen XI network depends on the *in vivo* loading history of the cultured limbs. We conclude therefore that mechanical loading from skeletal muscle contractions is likely to contribute to early events in establishment of the collagen XI network, even though the effects of fetal immobility on collagen XI do not become apparent until TS27.

## 3. Discussion

This study provides evidence for the role of muscle contraction-induced mechanical stimulation in promoting the correct spatial localization and structure of key cartilage and bone collagens over prenatal skeletal development. In the Splotch-delayed (muscleless-limb) model, spatial localizations of collagens I, II, V and X were aberrant, as were the structural organizations of collagens I, II, VI, X and XI. Effects on localization and structure varied over the three stages studied, with differences in localization being particularly pronounced at the earliest stage (TS22) and differences in structure becoming more pronounced with advancing development. We next tested the hypothesis that the effects of absent skeletal muscle on collagen structure and localization are predominantly due to the absence of muscle contraction-induced mechanical loading. Mechanical loading *in vitro* directly modulated the structure and localization of collagen II and the localization of collagen X. Collagen XI was not directly modulated by *in vitro* loading, which may be related to the age at which the limb explants were harvested. Finally, we investigated if mechanical loading *in vitro* could rescue any of the effects of fetal akinesia on collagen network emergence by culturing muscleless-limb explants*.* Mechanical loading *in vitro* rescued some features of the collagen network seen in the muscleless-limbs, including collagen II localization in the future articular cartilage region of the proximal humerus, collagen II structure in the proliferative region of the growth plate, and localization and structure of collagen X. We conclude therefore that skeletal muscle is essential for the normal development of the key cartilage and bone collagens and that mechanical loading from muscle contractions is directly involved in the establishment of the collagen II and X networks.

This work has shown that skeletal muscles are critical for the correct structural organization of key collagens in prenatal cartilage and bone. The importance of mechanical loading for collagen fibre organization has been previously demonstrated in postnatal and adult articular cartilage [5,47–53]. Collagen fibres align perpendicular to the loading direction in tissue-engineered cartilage constructs grown under unconfined compression [50–52]. Brama *et al*. [5] showed that high-intensity exercise in horses led to increased collagen parallelism and affected collagen orientation, with region-specific effects according to loading of individual joint sites. In hamsters, wheel-exercise led to altered collagen network organization and collagen content compared to non-exercised controls [49]. Changes in collagen content or organization were also found in wheel-exercised guinea pigs [48], while in dogs, alterations in collagen orientation but no changes in collagen content were found after an intense exercise programme [47]. The advance of the current study is that it demonstrates that skeletal muscles are essential for collagen fibre localization and structure in prenatal cartilage and bone, and also identifies which of the major skeletal collagen types are most and least sensitive to muscle loading.

Time-variant effects of muscle or loading were apparent for some collagens, particularly collagens II and XI. *In vivo*, absent skeletal muscle was associated with precocious formation of the collagen II mesh-like structure at early stages, which was lost by the latest stage examined. Our hypothesis is that muscle loading is a constraining factor for the early formation of collagen II structure, but that loading is needed to sustain healthy development of the network. Collagen XI was dramatically affected by absent muscle *in vivo* only at the latest stage of development studied, while collagen XI in limbs harvested at e15.5 was unaffected by mechanical loading *in vitro*. These data would indicate either that early establishment of the collagen XI network is independent of mechanical loading, or that the effects of absent loading do not become apparent until a later developmental stage. Our finding that the collagen XI network was more normal in mechanically stimulated muscleless-limbs compared to statically cultured muscleless-limbs indicates that the latter hypothesis is more likely, that early loading (prior to e15.5) of the limbs contributes to later collagen XI emergence. Moreover, collagen II is secreted in two isoforms into the matrix [54–56]. *Col2a1* is alternatively spliced during chondrogenesis, and the spliced variants are expressed differently by immature (IIA variant) and mature chondrocytes (IIB variant) and modulated differently by collagen XI. Perhaps an investigation of the collagen IIA variant and the interaction between IIA variant with collagen XI would have revealed the impact of a lack of mechanical stimulation on the organization of both collagen II and XI in the early stages of development.

Different levels of sensitivity to loading were exhibited in different regions of the developing rudiment, particularly *in vitro*. The effects of *in vitro* mechanical loading on collagens II and X were pronounced in the growth plate and future articular cartilage regions, but not in the epiphyseal (resting) cartilage. *In vitro* cultured limbs were harvested at e15.5, by which time the key events of epiphyseal cartilage collagen development have already initiated. We propose therefore that early stimulation of the limbs (prior to e15.5) is most important for initiation of the appropriate collagen network in the epiphyseal cartilage. In contrast to the epiphyseal cartilage, the prenatal growth plate requires sustained mechanical stimulation for proper development of its collagens, likely due to the dynamism of its development [57,58]. Given the importance of collagen X in the orchestration of chondrocyte hypertrophy [59], the recovery of restricted localization of collagen X within the growth plate in dynamically cultured muscleless-limbs may also imply that mechanostimulation is important in preventing premature chondrocyte differentiation and hypertrophy. The future articular cartilage region may also be undergoing dynamic processes in the chosen timeframe based on the observed changes in collagen II localization in the region between statically cultured and mechanically stimulated limbs. Mechanical loading has not yet been implicated in prenatal development of the future articular cartilage, despite the known importance of loading for postnatal articular cartilage [5,47–53], providing substantial scope for further investigation of the role of mechanical loading in directing articular cartilage development.

The effect of lack of muscles on the collagen localization and architecture also varied between samples, with one sample almost completely lacking collagen XI architecture. By contrast, minimal to no variation in collagen organization was seen between wild-type samples [9]. It is a well-established fact in the literature that there exists a high degree of inter-sample and intra-sample variability in the bones, joints and vertebrae of mice when skeletal muscle is either absent or non-contractile [18,19,23,24]. The inter-sample variability in collagen organization in the muscleless-limbs is thought to be caused by the embryos with muscle defects used in this study not experiencing complete fetal immobility *in utero*. In several of our other works, we have proposed that external mechanical loading due to movements of the dam and/or of the wild-type littermates affects development of the skeleton in muscleless-limb embryos. We have quantified the effects of such loading using computational modelling and have correlated differential effects of passive movements on the stresses and strains, and mechanosensitive gene expression, in the differentially affected rudiments of the muscleless-limb mouse embryo [60]. In our recent paper, we propose that the reduction in severity of shape and mineralization effects of absent skeletal muscle as development progresses may be due to increased external mechanical stimulation as the embryo grows [18]. Our theory of the importance of loading external to the muscleless-limb embryo is coherent with differences seen between pharmacologically immobilized chick embryos and muscleless-limb embryos. Immobilized chick embryos exhibit much more severe changes in skeletal development than mammalian models of altered fetal movements. Immobilized chick embryos experience no external loading, being isolated in the egg, in distinct contrast to mammalian embryos.

Our study is not without limitations. Tissue sections were used to characterize the collagen distribution and network architecture and, therefore, the three-dimensional organizations were not characterized. Combining tissue clearing techniques for immunofluorescence with light sheet microscopy would enable characterization of collagens in greater resolution and through a greater depth of the rudiment. Previous research has shown that mechanostimulation through muscle contraction is necessary for establishing proper chondrocyte volume [18], density [61] and organization [62]. Three-dimensional characterizations, especially the analysis of nearest neighbour relationships between chondrocytes and collagens using synchrotron-based imaging and light-sheet microscopy, would have allowed us to determine if disorganized chondrocyte organization contributes to the abnormal collagen architecture seen in the muscleless-limbs. Finally, statistical analyses were not feasible due to the small sample size, which was mainly due to the low breeding rates and therefore scarcity of muscleless embryos. However, studying multiple developmental stages, and both *in vivo* and *in vitro* loading scenarios, strengthens our conclusions.

The work presented here has provided novel insights into the mechanoregulation of the increasing complexity of the multiscale collagen network over skeletal development. We have demonstrated a direct role for mechanical loading for emergence of collagen distribution and structure during prenatal skeletal development. We have shown that collagens I, II, V, VI, X and XI are sensitive to the absence of skeletal muscles *in vivo*, and that collagens II and X are directly modulated by mechanical loading *in vitro*. These findings further our understanding of cartilage and bone matrix development, and in particular the effects of fetal akinesia on the collagen matrix of skeletal tissues. The finding that external mechanical loading can partially rescue the effects of fetal akinesia on the collagen matrix opens avenues towards physical therapies which could mitigate against the effects of reduced or abnormal fetal movements on skeletal development. Furthermore, the key to successful regeneration of bone and cartilage (which has so far remained elusive [63]) may be through understanding how the complexity arises over development, i.e. a developmentally inspired approach. The advance in understanding on the contribution of mechanical loading to collagen network establishment and maturation provided by this research will facilitate future advances in developmentally inspired replication of the collagen architecture in engineered skeletal tissues.

## 4. Material and methods

### 4.1. Tissue collection and processing

All experiments were performed in accordance with European legislation (Directive 2010/63/EU). For the immunofluorescence of collagens, and for the cultures of muscleless limbs, heterozygous *Splotch-delayed* (Pax3^spd/+^) females and males (imported from the Jackson Laboratory, Maine, USA; JAX stock no. 000565) were crossed to generate homozygous Pax3^Spd/Spd^ ‘muscleless limb’ embryos. Pax-3 is a mammalian homologue of the Drosophila paired gene which encodes a transcription factor and belongs to a family of developmentally regulated genes [64]. In the mouse, Pax-3 is expressed in the mesodermal and neuroectodermal derivatives. Mice homozygous for mutations in Pax-3^spd/spd^ have defects in the migration of muscle progenitor cells into the limb buds and consequently no skeletal muscle develops [65,66]. For limbs designated for immunofluorescence of collagens, embryos were harvested at e13.5, e16.5 and e18.5 and selected for analysis based on being categorized into Theiler stages TS22, TS25 or TS27 [32]. The wild-type limbs used for immunofluorescence of collagens were the same limbs described in our previous study [9]. For the wild-type explant cultures, embryos were harvested from pregnant C56Bl/6 J mice (Charles River, Massachusetts, USA; stock no. 3062894) at e15.5. For the muscleless limb cultures, Pax3^Spd/Spd^ muscleless limb embryos were harvested at e15.5. All embryos from the Splotch-delayed line were genotyped using PCR on DNA derived from head tissue. The PCR reaction was carried out for 30 cycles, each with duration of 30 s at 94°C, 60°C and 74°C, using three primers. The primer sequences used were AGGGCCGAGTCAACCAGCACG and CACGCGAAGCTGGCGAGAAATG for controls and AGTGTCCACCCCTCTTGGCCTCGGCCGAGTCAACCAGGTCC and CACGCGAAGCTGGCGAGAAATG for mutants. Sample numbers are detailed in tables 1 and 2.

**Table 1.** Numbers of limbs analysed for *in vivo* collagen characterization experiments.

collagen type	*in vivo*					
	TS22		TS25		TS27	
	wild-type	muscleless	wild-type	muscleless	wild-type	muscleless
I	3	3	3	2	4	3
II	4	3	3	2	3	3
III	2	3	2	2	2	1
V	3	3	3	2	3	3
VI	3	3	3	2	3	3
X	3	2	3	2	3	3
XI	3	2	3	1	3	3

**Table 2.** Numbers of limbs analysed for *in vitro* studies.

collagen type	bioreactor			
	wild-type		muscleless	
	static	dynamic	static	dynamic
II	3	3	3	3
X	2	2	3	3
XI	2	2	2	2

### 4.2. Immunofluorescence and image acquisition by confocal microscopy

Forelimbs were harvested and processed for cryosectioning. Forelimbs were chosen as they were most severely affected by the lack of skeletal muscles [18,23,24]. For cryoprotection, limbs were exposed to an increasing sucrose gradient (15% and 30% sucrose) and then embedded in optimal cutting temperature (Agar Scientific, Stansted, UK) diluted with 50% sucrose. Twelve millimetre thick sections were cut using a cryostat (NX70, Leica Biosystems, UK) [9]. For immunofluorescence, cryosections were permeabilized with 0.1% Tween 20 (Sigma-Aldrich)/1% dimethyl sulfoxide (DMSO; Sigma-Aldrich) in phosphate-buffered saline (PBS) and blocked with 5% (v/v) normal goat serum (Sigma-Aldrich). Following blocking, tissue sections were either treated with 10 mg ml^−1^ bovine testicular hyaluronidase (HA) (Sigma-Aldrich) for 1 h (collagens III and XI) prior to incubation with primary antibody or directly incubated with primary antibodies without HA treatment (collagens I, II, V, VI and X). Primary antibodies against specific collagen types were used in 1:50 dilutions. Following an 18 h incubation with the primary antibody at 4°C, tissues were washed and further incubated with a secondary antibody (1:200 dilutions) and 4′,6-diamidino-2-phenylindole (DAPI; Sigma-Aldrich) (1:2000 dilution) for 2 h at room temperature. Details of both the primary and secondary antibodies are provided in table 3. Direct fluorescence acquisition of labelled tissue sections was performed using an inverted confocal laser scanning microscope (Zeiss LSM 510 and Leica CF6) as described previously [9].

**Table 3.** List of antibodies used and their product details.

antibody	supplier	catalogue no.
collagen I	Abcam, UK	ab34710
collagen II	Sigma-Aldrich, UK	MAB8887
collagen III	Abcam, UK	ab7778
collagen V	Abcam, UK	ab7046
collagen VI	Abcam, UK	ab6588
collagen X	Abcam, UK	ab58632
collagen XI	Invitrogen, UK	PA5-77258
goat anti-rabbit (Cy3®)	Abcam, UK	ab6939
rabbit anti-mouse (Alexa Fluor® 488)	Abcam, UK	ab150125

### 4.3. Image analysis

Details of the number of limbs analysed for each experiment type are given in tables 1 and 2. Confocal images were processed in FIJI [33]. Whole rudiment images were created using Stitching (Pairwise stitching) plugin [67] in FIJI. Linear Blending was used as the method of Fusion and all channels were averaged for image registration. Where automatic stitching created blurry images in the overlapped zone, images were manually stitched in Inkscape (Inkscape Project, 2021, retrieved from https://inkscape.org).

### 4.4. Mechanostimulation bioreactor protocol

Following harvest at e15.5, the forelimbs were finely dissected and the soft tissues were manually removed with the forceps [68]. The forelimbs were then pinned at the scapula to a deformable foam base cut into rectangular steps as described previously [46]. Limbs designated for dynamic culture were then placed within the Ebers TC-3 bioreactor chambers (Don Whitley Scientific, Bingley, UK) to undergo uniaxial compression. Contralateral limbs designated for static culture were placed (with the same foam support as the dynamic cultures) in a Petri dish. Limb explants were cultured at an air–liquid interface using basal media (α-MEM GlutaMAX, Thermo Fisher Scientific, USA) supplemented with 1% penicillin–streptomycin with amophotericin B, 100 µM ascorbic acid (Sigma-Aldrich, USA), 2 mM β-glycerophosphate and 100 nM dexamethasone. Explant cultures were incubated for 6 days at 37°C in a humidified atmosphere containing 5% CO_2_. An air–liquid interface was used to culture the limb explants, and the medium was changed every 24 h to keep the tissues from drying out and to make sure the limbs were always halfway submerged. Limb explants designated for dynamic culture were subjected to mechanical deformation. The loading regime comprised cyclical compression of the forelimbs at 0.67 Hz to a displacement of 2 mm for 2 h, three times per day. This produced approximately 14° angle cyclic flexion of the elbow. The angle was estimated based on previous studies carried out on chick joints using the same experimental set-up [46]. Paired comparisons of collagens were performed between the mechanically loaded forelimb and the contralateral statically cultured forelimb, from the same embryo.

## Data Availability

All data are available in the main text or the electronic supplementary material [69].

## References

[1] Myllyharju J, Kivirikko KI. 2001 Collagens and collagen-related diseases. Ann. Med. **33**, 7-21. (10.3109/07853890109002055) 11310942

[2] Basel D, Steiner RD. 2009 Osteogenesis imperfecta: recent findings shed new light on this once well-understood condition. Genet. Med. **11**, 375-385. (10.1097/GIM.0b013e3181a1ff7b) 19533842

[3] Warman ML, Abbott M, Apte SS, Hefferon T, McIntosh I, Cohn DH, Hecht JT, Olsen BR, Francomano CA. 1993 A type X collagen mutation causes Schmid metaphyseal chondrodysplasia. Nat. Genet. **5**, 79-82. (10.1038/ng0993-79) 8220429

[4] Rozario T, DeSimone DW. 2010 The extracellular matrix in development and morphogenesis: a dynamic view. Dev. Biol. **341**, 126-140. (10.1016/j.ydbio.2009.10.026) 19854168 PMC2854274

[5] Brama PA, Holopainen J, van Weeren PR, Firth EC, Helminen HJ, Hyttinen MM. 2009 Effect of loading on the organization of the collagen fibril network in juvenile equine articular cartilage. J. Orthop. Res. **27**, 1226-1234. (10.1002/jor.20866) 19242977

[6] Hunziker EB, Kapfinger E, Geiss J. 2007 The structural architecture of adult mammalian articular cartilage evolves by a synchronized process of tissue resorption and neoformation during postnatal development. Osteoarthritis Cartilage **15**, 403-413. (10.1016/j.joca.2006.09.010) 17098451

[7] Li L et al. 2017 Superficial cells are self-renewing chondrocyte progenitors, which form the articular cartilage in juvenile mice. FASEB J. **31**, 1067-1084. (10.1096/fj.201600918R) 27965322 PMC5295727

[8] Decker RS et al. 2017 Cell origin, volume and arrangement are drivers of articular cartilage formation, morphogenesis and response to injury in mouse limbs. Dev. Biol. **426**, 56-68. (10.1016/j.ydbio.2017.04.006) 28438606 PMC6046638

[9] Ahmed S, Nowlan NC. 2020 Initiation and emerging complexity of the collagen network during prenatal skeletal development. Eur. Cell Mater. **39**, 136-155. (10.22203/eCM.v039a09) 32103474

[10] Klein-Nulend J, Bacabac RG, Bakker AD. 2012 Mechanical loading and how it affects bone cells: the role of the osteocyte cytoskeleton in maintaining our skeleton. Eur. Cell Mater. **24**, 278-291. (10.22203/ecm.v024a20) 23007912

[11] Miller ME, Hangartner TN. 1999 Temporary brittle bone disease: association with decreased fetal movement and osteopenia. Calcif. Tissue Int. **64**, 137-143. (10.1007/s002239900592) 9914321

[12] Hall JG. 1986 Analysis of Pena Shokeir phenotype. Am. J. Med. Genet. **25**, 99-117. (10.1002/ajmg.1320250112) 3541610

[13] Hammond E, Donnenfeld AE. 1995 Fetal akinesia. Obstet. Gynecol. Surv. **50**, 240-249. (10.1097/00006254-199503000-00028) 7739837

[14] Nicole S, Diaz CC, Frugier T, Melki J. 2002 Spinal muscular atrophy: recent advances and future prospects. Muscle Nerve **26**, 4-13. (10.1002/mus.10110) 12115944

[15] Wesström G, Bensch J, Schollin J. 1986 Congenital myotonic dystrophy. Incidence, clinical aspects and early prognosis. Acta Paediatr. Scand. **75**, 849-854. (10.1111/j.1651-2227.1986.tb10301.x) 3564952

[16] Rodríguez JI, Garcia-Alix A, Palacios J, Paniagua R. 1988 Changes in the long bones due to fetal immobility caused by neuromuscular disease. A radiographic and histological study. J. Bone Joint Surgery Am. **70**, 1052-1060. (10.2106/00004623-198870070-00014) 3403574

[17] Rodríguez JI, Palacios J, García-Alix A, Pastor I, Paniagua R. 1988 Effects of immobilization on fetal bone development. A morphometric study in newborns with congenital neuromuscular diseases with intrauterine onset. Calcif. Tissue Int. **43**, 335-339. (10.1007/bf02553275) 3146421

[18] Sotiriou V, Huang Y, Ahmed S, Isaksson H, Nowlan NC. 2022 Prenatal murine skeletogenesis partially recovers from absent skeletal muscle as development progresses. Eur. Cell Mater. **44**, 115-132. (10.22203/eCM.v044a08) 36345651

[19] Levillain A, Ahmed S, Kaimaki DM, Schuler S, Barros S, Labonte D, Iatridis JC, Nowlan NC. 2021 Prenatal muscle forces are necessary for vertebral segmentation and disc structure, but not for notochord involution in mice. Eur. Cell Mater. **41**, 558-575. (10.22203/eCM.v041a36) 34021906 PMC8268087

[20] Levillain A, Rolfe RA, Huang Y, Iatridis JC, Nowlan NC. 2019 Short-term foetal immobility temporally and progressively affects chick spinal curvature and anatomy and rib development. Eur. Cell Mater. **37**, 23-41. (10.22203/eCM.v037a03) 30644077 PMC6505690

[21] Bridglal DL, Boyle CJ, Rolfe RA, Nowlan NC. 2021 Quantifying the tolerance of chick hip joint development to temporary paralysis and the potential for recovery. Dev. Dynamics **250**, 450-464. (10.1002/dvdy.236) 32776603

[22] Brunt LH, Skinner RE, Roddy KA, Araujo NM, Rayfield EJ, Hammond CL. 2016 Differential effects of altered patterns of movement and strain on joint cell behaviour and skeletal morphogenesis. Osteoarthritis Cartilage **24**, 1940-1950. (10.1016/j.joca.2016.06.015) 27374878 PMC5081689

[23] Kahn J et al. 2009 Muscle contraction is necessary to maintain joint progenitor cell fate. Dev. Cell **16**, 734-743. (10.1016/j.devcel.2009.04.013) 19460349

[24] Nowlan NC, Bourdon C, Dumas G, Tajbakhsh S, Prendergast PJ, Murphy P. 2010 Developing bones are differentially affected by compromised skeletal muscle formation. Bone **46**, 1275-1285. (10.1016/j.bone.2009.11.026) 19948261 PMC2860222

[25] Nowlan NC, Chandaria V, Sharpe J. 2014 Immobilized chicks as a model system for early-onset developmental dysplasia of the hip. J. Orthop. Res. **32**, 777-785. (10.1002/jor.22606) 24590854

[26] Nowlan NC, Sharpe J. 2014 Joint shape morphogenesis precedes cavitation of the developing hip joint. J. Anat. **224**, 482-489. (10.1111/joa.12143) 24266523 PMC4098681

[27] Khatib N, Parisi C, Nowlan NC. 2021 Differential effect of frequency and duration of mechanical loading on fetal chick cartilage and bone development. Eur. Cell Mater. **41**, 531-545. (10.22203/eCM.v041a34) 34033115

[28] Sharir A, Stern T, Rot C, Shahar R, Zelzer E. 2011 Muscle force regulates bone shaping for optimal load-bearing capacity during embryogenesis. Development **138**, 3247-3259. (10.1242/dev.063768) 21750035

[29] Benninghoff A. 1925 Form und Bau der Gelenkknorpel in ihren Beziehungen zur Funktion. Zeitschrift für Zellforschung und mikroskopische Anatomie **2**, 783-862. (10.1007/BF00583443)

[30] Gründer W. 2006 MRI assessment of cartilage ultrastructure. NMR Biomed. **19**, 855-876. (10.1002/nbm.1092) 17075962

[31] Kalson NS, Starborg T, Lu Y, Mironov A, Humphries SM, Holmes DF, Kadler KE. 2013 Nonmuscle myosin II powered transport of newly formed collagen fibrils at the plasma membrane. Proc. Natl Acad. Sci. USA **110**, E4743. (10.1073/pnas.1314348110) 24248360 PMC3856828

[32] Theiler K. 1989 The house mouse: atlas of embryonic development. Berlin, Germany: Springer. http://link.springer.com/10.1007/978-3-642-88418-4.

[33] Schindelin J et al. 2012 Fiji: an open-source platform for biological-image analysis. Nat. Methods. **9**, 676-682. (10.1038/nmeth.2019) 22743772 PMC3855844

[34] Viguet-Carrin S, Garnero P, Delmas PD. 2006 The role of collagen in bone strength. Osteoporosis Int. **17**, 319-336. (10.1007/s00198-005-2035-9) 16341622

[35] Aszodi A, Chan D, Hunziker E, Bateman JF, Fassler R. 1998 Collagen II is essential for the removal of the notochord and the formation of intervertebral discs. J. Cell Biol. **143**, 1399-1412. (10.1083/jcb.143.5.1399) 9832566 PMC2133086

[36] Li SW et al. 1995 Transgenic mice with targeted inactivation of the Col2 alpha 1 gene for collagen II develop a skeleton with membranous and periosteal bone but no endochondral bone. Genes Dev. **9**, 2821-2830. (10.1101/gad.9.22.2821) 7590256

[37] Beighton P, De Paepe A, Steinmann B, Tsipouras P, Wenstrup RJ. 1998 Ehlers-Danlos syndromes: revised nosology, Villefranche, 1997. Am. J. Med. Genet. **77**, 31-37. (10.1002/(SICI)1096-8628(19980428)77:1<31::AID-AJMG8>3.0.CO;2-O) 9557891

[38] Birk DE. 2001 Type V collagen: heterotypic type I/V collagen interactions in the regulation of fibril assembly. Micron (Oxford, England: 1993) **32**, 223-237. (10.1016/S0968-4328(00)00043-3) 11006503

[39] Glimcher MJ, Shapiro F, Ellis RD, Eyre DR. 1980 Changes in tissue morphology and collagen composition during the repair of cortical bone in the adult chicken. J. Bone Joint Surgery Am. **62**, 964-973. (10.2106/00004623-198062060-00013) 7430185

[40] Niyibizi C, Eyre DR. 1989 Bone type V collagen: chain composition and location of a trypsin cleavage site. Connect. Tissue Res. **20**, 247-250. (10.3109/03008208909023894) 2612158

[41] Zelenski NA, Leddy HA, Sanchez-Adams J, Zhang J, Bonaldo P, Liedtke W, Guilak F. 2015 Type VI collagen regulates pericellular matrix properties, chondrocyte swelling, and mechanotransduction in mouse articular cartilage. Arthritis Rheum. **67**, 1286-1294. (10.1002/art.39034) PMC4414817 25604429

[42] Alexopoulos LG, Youn I, Bonaldo P, Guilak F. 2009 Developmental and osteoarthritic changes in Col6a1-knockout mice: biomechanics of type VI collagen in the cartilage pericellular matrix. Arthritis Rheum. **60**, 771-779. (10.1002/art.24293) 19248115 PMC2724839

[43] Sweeney E, Roberts D, Corbo T, Jacenko O. 2010 Congenic mice confirm that collagen X is required for proper hematopoietic development. PLoS ONE **5**, e9518. (10.1371/journal.pone.0009518) 20209091 PMC2831078

[44] Li A, Wei Y, Hung C, Vunjak-Novakovic G. 2018 Chondrogenic properties of collagen type XI, a component of cartilage extracellular matrix. Biomaterials **173**, 47-57. (10.1016/j.biomaterials.2018.05.004) 29758546

[45] Wu JJ, Weis MA, Kim LS, Eyre DR. 2010 Type III collagen, a fibril network modifier in articular cartilage. J. Biol. Chem. **285**, 18 537-18 544. (10.1074/jbc.M110.112904) 20404341 PMC2881779

[46] Chandaria VV, McGinty J, Nowlan NC. 2016 Characterising the effects of in vitro mechanical stimulation on morphogenesis of developing limb explants. J. Biomech. **49**, 3635-3642. (10.1016/j.jbiomech.2016.09.029) 27743631 PMC5765238

[47] Arokoski JP, Hyttinen MM, Lapveteläinen T, Takács P, Kosztáczky B, Módis L, Kovanen V, Helminen H. 1996 Decreased birefringence of the superficial zone collagen network in the canine knee (stifle) articular cartilage after long distance running training, detected by quantitative polarised light microscopy. Ann. Rheum. Dis. **55**, 253-264. (10.1136/ard.55.4.253) 8733443 PMC1010147

[48] Hyttinen MM, Arokoski JP, Parkkinen JJ, Lammi MJ, Lapveteläinen T, Mauranen K, Király K, Tammi MI, Helminen HJ. 2001 Age matters: collagen birefringence of superficial articular cartilage is increased in young guinea-pigs but decreased in older animals after identical physiological type of joint loading. Osteoarthritis Cartilage **9**, 694-701. (10.1053/joca.2001.0466) 11795988

[49] Julkunen P et al. 2010 Effects of growth and exercise on composition, structural maturation and appearance of osteoarthritis in articular cartilage of hamsters. J. Anat. **217**, 262-274. (10.1111/j.1469-7580.2010.01270.x) 20646109 PMC2972540

[50] Kelly TA, Ng KW, Wang CC, Ateshian GA, Hung CT. 2006 Spatial and temporal development of chondrocyte-seeded agarose constructs in free-swelling and dynamically loaded cultures. J. Biomech. **39**, 1489-1497. (10.1016/j.jbiomech.2005.03.031) 15990101

[51] Lee EJ, Holmes JW, Costa KD. 2008 Remodeling of engineered tissue anisotropy in response to altered loading conditions. Ann. Biomed. Eng. **36**, 1322-1334. (10.1007/s10439-008-9509-9) 18470621 PMC2920500

[52] Martínez H, Brackmann C, Enejder A, Gatenholm P. 2012 Mechanical stimulation of fibroblasts in micro-channeled bacterial cellulose scaffolds enhances production of oriented collagen fibers. J. Biomed. Mater. Res. A **100**, 948-957. (10.1002/jbm.a.34035) 22275210

[53] Nakatsuji N, Johnson KE. 1984 Experimental manipulation of a contact guidance system in amphibian gastrulation by mechanical tension. Nature **307**, 453-455. (10.1038/307453a0) 6694737

[54] Hering TM, Wirthlin L, Ravindran S, McAlinden A. 2014 Changes in type II procollagen isoform expression during chondrogenesis by disruption of an alternative 5’ splice site within Col2a1 exon 2. Matrix Biol. **36**, 51-63. (10.1016/j.matbio.2014.04.004) 24735995 PMC4323166

[55] Lewis R, Ravindran S, Wirthlin L, Traeger G, Fernandes RJ, McAlinden A. 2012 Disruption of the developmentally-regulated Col2a1 pre-mRNA alternative splicing switch in a transgenic knock-in mouse model. Matrix Biol. **31**, 214-226. (10.1016/j.matbio.2011.12.004) 22248926 PMC3295890

[56] McAlinden A. 2014 Alternative splicing of type II procollagen: IIB or not IIB? Connect. Tissue Res. **55**, 165-176. (10.3109/03008207.2014.908860) 24669942 PMC4317353

[57] Lamuedra A, Gratal P, Calatrava L, Ruiz-Perez VL, Largo R, Herrero-Beaumont G. 2020 Disorganization of chondrocyte columns in the growth plate does not aggravate experimental osteoarthritis in mice. Sci. Rep. **10**, 10745. (10.1038/s41598-020-67518-0) 32612184 PMC7329885

[58] Lee D, Erickson A, Dudley AT, Ryu S. 2019 Mechanical stimulation of growth plate chondrocytes: previous approaches and future directions. Exp. Mech. **59**, 1261-1274. (10.1007/s11340-018-0424-1) 31787777 PMC6884322

[59] Irwin MH, Silvers SH, Mayne R. 1985 Monoclonal antibody against chicken type IX collagen: preparation, characterization, and recognition of the intact form of type IX collagen secreted by chondrocytes. J. Cell Biol. **101**, 814-823. (10.1083/jcb.101.3.814) 2411737 PMC2113712

[60] Nowlan NC, Dumas G, Tajbakhsh S, Prendergast PJ, Murphy P. 2012 Biophysical stimuli induced by passive movements compensate for lack of skeletal muscle during embryonic skeletogenesis. Biomech. Model. Mechanobiol. **11**, 207-219. (10.1007/s10237-011-0304-4) 21505895 PMC4794622

[61] Pierantoni M, Le Cann S, Sotiriou V, Ahmed S, Bodey AJ, Jerjen I, Nowlan NC, Isaksson H. 2021 Muscular loading affects the 3D structure of both the mineralized rudiment and growth plate at early stages of bone formation. Bone **145**, 115849. (10.1016/j.bone.2021.115849) 33454374

[62] Shwartz Y, Farkas Z, Stern T, Aszódi A, Zelzer E. 2012 Muscle contraction controls skeletal morphogenesis through regulation of chondrocyte convergent extension. Dev. Biol. **370**, 154-163. (10.1016/j.ydbio.2012.07.026) 22884393

[63] Walter SG, Ossendorff R, Schildberg FA. 2019 Articular cartilage regeneration and tissue engineering models: a systematic review. Arch. Orthop. Trauma Surg. **139**, 305-316. (10.1007/s00402-018-3057-z) 30382366

[64] Strachan T, Read AP. 1994 PAX genes. Curr. Opin. Genet. Dev. **4**, 427-438. (10.1016/0959-437x(94)90032-9) 7919921

[65] Franz T, Kothary R, Surani MA, Halata Z, Grim M. 1993 The Splotch mutation interferes with muscle development in the limbs. Anatomy Embryol. **187**, 153-160. (10.1007/bf00171747) 8238963

[66] Tajbakhsh S, Rocancourt D, Cossu G, Buckingham M. 1997 Redefining the genetic hierarchies controlling skeletal myogenesis: Pax-3 and Myf-5 act upstream of MyoD. Cell **89**, 127-138. (10.1016/s0092-8674(00)80189-0) 9094721

[67] Preibisch S, Saalfeld S, Tomancak P. 2009 Globally optimal stitching of tiled 3D microscopic image acquisitions. Bioinformatics **25**, 1463-1465. (10.1093/bioinformatics/btp184) 19346324 PMC2682522

[68] Henstock JR, Rotherham M, Rose JB, El Haj AJ. 2013 Cyclic hydrostatic pressure stimulates enhanced bone development in the foetal chick femur in vitro. Bone **53**, 468-477. (10.1016/j.bone.2013.01.010) 23333177

[69] Ahmed S, Rogers AV, Nowlan NC. 2023 Mechanical loading due to muscle movement regulates establishment of the collagen network in the developing murine skeleton. *Figshare*. (10.6084/m9.figshare.c.6858288) PMC10582611 37859832

